# Halfway Through Ex Situ Population Genetic Lifespan: The Case of *Cochlearia polonica*

**DOI:** 10.3390/biology14060681

**Published:** 2025-06-11

**Authors:** Anna Rucińska, Katarzyna Joanna Chwedorzewska, Piotr Tomasz Bednarek, Maja Boczkowska, Jerzy Puchalski, Piotr Androsiuk, Ewa Czaplicka

**Affiliations:** 1 Polish Academy of Sciences Botanical Garden—Center for Biological Diversity Conservation in Powsin, Prawdziwka 2, 02-973 Warsaw, Poland; jtpuchalski@wp.pl (J.P.);; 2 Plant Breeding and Acclimatization Institute-National Research Institute, 05-870 Błonie, Poland; p.bednarek@ihar.edu.pl (P.T.B.); m.boczkowska@ihar.edu.pl (M.B.); 3 Department of Botany, Warsaw University of Life Sciences-SGGW, Nowoursynowska 159, 02-776 Warsaw, Poland; katarzyna_chwedorzewska@sggw.edu.pl; 4 Department of Plant Physiology, Genetics and Biotechnology, Faculty of Biology and Biotechnology, University of Warmia and Mazury in Olsztyn, M. Oczapowskiego 1A, 10-719 Olsztyn, Poland; piotr.androsiuk@uwm.edu.pl

**Keywords:** ex situ conservation, botanical garden, conservation genetics, bottleneck effect, threatened species, endemic species

## Abstract

Although the usage of genetic data for the conservation management of threatened species is growing rapidly, the assessment of the trajectory and persistence of ex situ and in situ populations suffers from a lack of monitoring of genetic indicators. To fill this gap, we conducted a comparative analysis of the genetic structure ex situ and its source population of *Cochlearia polonica* to improve conservation outcomes for this species. *C. polonica*, as a range-restricted endemic species in Poland, exists in the wild in a single reintroduced population, which was the source for establishing an ex situ population in the botanical garden, and both populations have been well studied in abundance time series until the extinction of artificial populations due to genetic constraints. We collected AFLP data from individuals sampled after the peak recovery phase following the founder effect in the ex situ population, as well as from the source population, to assess the genetic consequences of long-term cultivation. The genetic profile of both populations suggested their strong differentiation. The genetic variation in the ex situ population expressed by Shannon’s Information Index and expected heterozygosity (He) was approximately half that of the source population. While ex situ population growth was high, the gene pool was homogenised, and genetic diversity waned, leading to its extinction across merely 5–6 generations. Thus, the only reintroduced population of *C. polonica* left in the wild is more threatened with extinction than previously thought, and conservation action should be taken immediately.

## Simple Summary

*Cochlearia polonica* is a critically endangered plant species naturally occurring in a limited area of Poland. After its original habitat was destroyed, conservationists established a cultivated population in a PAS Botanical Garden CBDC. This study compares the genetic profile of the last remaining wild population and the now extinct cultivated population. Although the cultivated group initially increased in number, it experienced a significant loss of genetic variation over a short time. The analysis reveals a divergence in genetic structure between the two populations, despite their shared origin. These results demonstrate the risks associated with cultivating endangered species outside their natural environment, especially when the starting population is small. Reduced genetic diversity can limit a species’ ability to adapt and survive. The findings emphasise the importance of maintaining genetic health in conservation efforts. To prevent further decline, it is essential to collect seeds from the remaining wild population and store them under secure, long-term conditions. This strategy could help conserve the species and support future restoration efforts.

## 1. Introduction

Endemic plant species, restricted to specific geographic regions, represent a unique and irreplaceable component of global biodiversity [1]. Due to their narrow ecological amplitude and localised distributions, they often evolve specialised traits that contribute to ecosystem stability and function [2]. However, these same characteristics make endemic species particularly vulnerable to habitat fragmentation, environmental change, and other anthropogenic pressures [3]. Consequently, endemic plants are overrepresented among globally threatened taxa, especially in biodiversity hotspots [4]. Studying and conserving this species is therefore critical not only for preserving regional floristic diversity but also for maintaining evolutionary processes and ecosystem resilience [5,6]. Conservation approaches that include both in situ and ex situ efforts offer valuable frameworks for mitigating extinction risks in these often-overlooked yet ecologically significant taxa.

Human activities such as land-use change, deforestation, urbanisation, overexploitation of natural resources, environmental pollution, and the introduction and spread of invasive alien species have threatened the existence of approximately one-third of the world’s 300,000–450,000 vascular plant species [7,8]. Protecting a threatened species in its natural habitat is the most effective remedy against extinction. However, if rapid habitat loss occurs, protection is impossible or hard to achieve [9]. The highest priority is to conserve species in their natural habitats (in situ), where they may experience the full range of interactions with various components of the biotic and abiotic environment and where the natural process of evolution can continue. Complementary protection efforts outside a species’ natural habitat (ex situ conservation) are also crucial for plant diversity conservation [10]. It is particularly essential for species when in situ threats are high. In many cases, it mitigates the extinction risk of species [11]. Ex situ methods include a set of techniques that depend on the type of plant material to be conserved and the facilities available at the conservation institution. Seeds and tissue cultures are usually preserved in fridges, cryotanks, or chambers, while living plant collections have gained the most attention from conservation botanists due to cultivation traditions in botanical gardens [12]. However, regardless of the conservation technique used, due to the high cost, labour intensity, and vast number of accessions preserved in such institutions, conservation programs carry a high risk of unfavourable demographic and genetic processes, which may lead to a reduction in the population size and, as a result, an increase in the probability of population extinction [8,13].

Ex situ living plant populations face genetic challenges similar to those encountered by small wild populations [14]. Both theoretical models and empirical evidence indicate that founder effects, genetic drift, and inbreeding can lead to reduced genetic diversity, particularly in small and isolated populations [15,16]. Species’ vulnerability to genetic erosion from a reduced population size and isolation depends on traits like the growth form and mating system, with short-lived herbaceous plants more affected than long-lived woody species [16,17].

One of the rarest species in the European flora is *Cochlearia polonica*. Only two species represent the genus *Cochlearia* (Brassicaceae) in Poland: *Cochlearia tatrae* Borbás, a narrow-endemic species restricted to the Tatra Mountains [18], and *C. polonica* Fröhl., which is currently present only in one secondary site near Olkusz, confined there to shallow water on the sandy ground of the river Centuria [19]. *Cochlearia* L. was first reported by Zalewski in 1886 in the Silesian-Cracow Upland near Olkusz. The population covered a large area in the shallow water of the Biała River. The plant was incorrectly identified as *Cochlearia officinalis* L. This finding was confirmed by Piech (1924) [20], who also classified it as a subspecies of *Cochlearia officinalis* L. subsp. *pyrenaica*. Later, Frölich [21] recognised it as a new species and named *it Cochlearia polonica* E. Frölich based on detailed morphological studies, which was then confirmed by Bajer’s cytological studies [22]. This study also suggested that the autopolyploidisation evolutionary process produced this species. Further analysis confirmed the polyploid genome of *C. polonica* [23,24].

**Figure 1:**
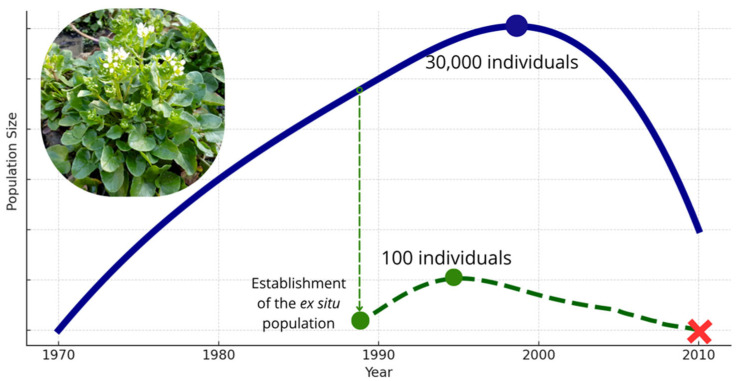
Symbolic demographic trajectories of seminatural and ex situ populations of *Cochlearia polonica* (1970–2010). The dark blue line illustrates the reconstructed population size of the seminatural population established in the Centuria locality, showing rapid growth after its introduction in 1970 and decline beginning after 2000. The dashed dark green line represents the ex situ population established in the late 1980s in the PAS Botanical Garden in Powsin, with a sharp increase up to 1994 followed by a gradual decline until extinction in 2010 (red cross).

The drainage of the species’ native range area, which began in the 1960s, led to the complete disappearance of the water network in the upper Biała River basin, which ultimately resulted in the destruction of the site [25], leading to population decay over two decades. In 1970, approximately a dozen plants were transplanted into a site near the Centuria River springs to establish a new population. The number of individuals in the transplanted population increased to approximately 30,000 individuals and spread over an area of 2000 m^2^ [25]. In the 1990s, further attempts were made to reintroduce the species, mainly in the Silesian-Cracow Upland, but without much success, as plants disappeared a few years after introduction [25,26]. Recent demographic data indicate that the population has decreased to 1500 individuals in the Centuria locality [27]. Currently, *C. polonica* is considered extinct in the wild, and the population at a secondary site is critically endangered. Finally, it was listed among the species with the highest conservation concerns (global IUCN Red List, Annexes II and IV of the EU Habitat Directive, Bern Convention; [25]). The ex situ population was founded with only a few individuals in the late 1980s in the PAS Botanical Garden CBDC in Powsin (Figure 1). By the end of 1994, the population made a remarkable recovery after a severe population bottleneck [28], reaching approximately 100 individuals, and then it showed approximately ten years of ongoing decline to complete extinction (personal observation). The ex situ population was cultivated under stable and uniform conditions throughout its entire lifespan, with consistent efforts to provide optimal settings for unrestricted plant growth. Given the absence of environmental variation, it is likely that the observed decline was primarily driven by genetic factors.

*Cochlearia polonica* populations offer a valuable model for exploring key concepts in conservation genetics, particularly those related to small population dynamics. Its documented history of habitat loss, demographic bottlenecks, and ex situ cultivation highlights processes such as a reduced effective population size, genetic drift, and inbreeding. Retrospective data allow for the examination of the ex situ population at a mid-point in its demographic trajectory, offering a rare insight into temporal shifts in genetic structure.

It also provides an opportunity to investigate several key aspects of genetic change in small populations and enables an assessment of the level of genetic diversity preserved in the ex situ population at the midpoint of its demographic trajectory, along with a comparison of genetic differentiation between the ex situ collection and the wild population. Together, these insights can contribute to a better understanding of the genetic profile ex situ and its source population.

It also relates to the long-standing challenge of developing strategies that, on the one hand, prevent or minimise genetic and demographic risks associated with the establishment of artificial populations [29,30] and, on the other hand, ensure the effective provision of material for in situ recovery efforts [31,32]. Thus, ex situ conservation is an essential component of conservation programs, and plant collection strategies need to ensure that the material sufficiently represents the diversity of the source populations [33,34].

Botanic gardens have heightened their conservation mission in recent years, providing plant material for ecological restoration to complement in situ efforts for the conservation of plant diversity [35], with a particular emphasis placed on the conservation of threatened plant species [36]. Most plants are conserved in botanical gardens as living plant collections [8,37,38,39]. There are more than 3600 botanical gardens and arboreta worldwide [40], with more than 150,000 species, 6 million accessions, and approximately 80,000 taxa, thousands of which are threatened with extinction [8,41]. However, the genetic diversity and structure of conservation priority taxa held as ex situ accessions have rarely been studied [8,41,42,43,44,45], creating a significant gap in the field of molecular ecology and conservation genetics.

To address these knowledge gaps, we assess patterns of genetic diversity of ex situ and in situ populations of *Cochlearia polonica* to construct an evidence-based conservation strategy for this endemic species. To do so, this study addresses the following questions: (i) What was the level of genetic diversity of the ex situ population in the middle of its lifespan? (ii) How differentiated were the populations from ex situ collection in the botanical garden and the natural stand in the Centuria River? (iii) What is the usefulness of genetic indices to monitor the effect of a bottleneck and mutation drift of two existing populations?

## 2. Materials and Methods

Two *C. polonica* populations were investigated: a population from the spring area of Centuria River (C; called the seminatural population in this study; 50°25′04″ N, 19°29′29″ E) and an ex situ population from the Polish Academy of Sciences Botanical Garden Center for Biological Diversity Conservation in Powsin—PAS Botanical Garden CBDC (BG; called ex situ or botanic garden population; 52°06′22″ N, 21°05′57″ E). A total of 36 individuals from the ex situ population (PAS Botanical Garden CBDC, Powsin) and 38 individuals from the seminatural population (Centuria locality) were sampled for genetic analysis in 2001. For each individual, approximately 100 mg of fresh leaf tissue was collected and immediately flash-frozen in liquid nitrogen in the field to preserve DNA integrity prior to extraction. According to the manufacturer’s procedure, total genomic DNA was extracted from each sample of fresh leaves (Qiagen, Hilden, Germany: DNeasy Plant Mini Kit). The purity and quantity of DNA were determined spectrophotometrically. The DNA integrity was tested in 1.4% agarose gels (1xTBE buffer and ethidium bromide—0.5 µg/mL, at 20 V/cm). DNA was stored in the DNA Bank (−80 °C) in the PAS Botanical Garden until further analysis.

The AFLP technique was performed according to the original procedure [46], following [47], with a minor modification using *Eco*RI/*Mse*I (New England Biolabs, Ipswich, MA, USA) enzymes to digest 500 ng of genomic DNA. For oligonucleotide adapter ligation, 10 μL of a mix containing 8 U of T4 DNA ligase New England Biolabs, Ipswich, MA, USA), 1× ligation buffer, 0.5 μM of NaCl, 2 nM of *Eco*RI adapter, and 0.2 μM of *Mse*I adapter was added to each digested sample. Ligation reactions were incubated for 2 hs at 37 °C. Adapter and primer sequences are listed in Table S2. Pre-selective amplification was performed using primers with a single selective nucleotide at the 3′ end (Table S2). Each 6 μL reaction consisted of 4.0 μL of pre-selective primer mix, 0.6 μL of 10× Taq polymerase buffer, 0.2 μL of Taq DNA polymerase (5 U/μL, Qiagen, Hilden, Germany), and 1.0 μL of diluted ligated DNA. The PCR cycling program included 20 cycles of 30 s at 94 °C, 60 s at 56 °C, and 60 s at 74 °C. Following amplification, the PCR product was diluted tenfold and used as a template for the selective amplification step, which was carried out using primers with two additional selective nucleotides at the 3′ end (Table S2). The 10 μL reaction mixture contained 0.25 μL of ^33P-labeled EcoRI primer, 2.25 μL of MseI primer (15.075 ng), 0.125 μL of Taq DNA polymerase (5 U/μL, Qiagen, Hilden, Germany), 1.0 μL of 10× buffer, 3.0 μL of diluted pre-selective PCR product, and 3.375 μL of ddH_2_O. The selective amplification protocol included a touchdown phase of 10 cycles with a decreasing annealing temperature, starting at 65 °C and ending at 56 °C (94 °C for 60 s, 65–56 °C for 60 s, and 72 °C for 90 s), followed by 23 cycles of standard amplification (94 °C for 30 s, 56 °C for 30 s, and 72 °C for 60 s).

Selective amplification was carried out in the presence of 5′-(^32^P)-labelled primers. Initially, sixty-four different primer pair combinations were tested. Among those combinations, eight primer pairs (E-AAA/M-CAG, E-AAA/M-CAT, E-AAC/E-CGT, E-AAC/M-CGA, E-AAG/M-CGA, E-AAG/M-CCG, E-AAT/M-CAC, E-AAT/M-CTG) were selected for the main study. Independent amplifications were run with each selected primer pair, and PCR products were separated on a 5% polyacrylamide gel and visualised by exposure to X-ray films at −70 °C overnight. Reproducible, clearly distinguishable bands were scored across all samples as either present (1) or absent (0) and recorded as a binary matrix. AFLPop 1.2 EXCEL add-in software [48] eliminated redundant markers (loci that show identical scores among all individuals across both populations).

GenAlEx 6.5 [49,50] was used to evaluate the following characteristics: allele frequencies; the number of bands shared among individuals with a frequency greater than or equal to 5%; the number of unique bands; percentage of polymorphic bands (P%); Shannon’s Information Index (I); and expected heterozygosity (He) for each population from binary data assuming Hardy–Weinberg equilibrium [51,52]. Comparisons between the ex situ and seminatural populations were conducted using two-tailed permutation tests (10,000 iterations) to evaluate the statistical significance of observed differences. To estimate genetic relationships between studied populations, Φ*_PT_*, the Nei genetic distance, and the Nei unbiased genetic identity were also estimated in GenAlEx 6.5. Arlequin software, version 3.11 [53], was used to analyse molecular variance (AMOVA) and estimate the F*_ST_* value. The significance of fixation indices was tested using a nonparametric permutation approach [54]. Arlequin version 3.11 was also used to estimate Tajima’s D, Fu’s F*_S_* neutrality, and the mismatch distribution and demographic processes affecting the populations [55].

The population structure was analysed using the Bayesian model-based clustering method implemented in STRUCTURE ver. 2.3.4. [56]. The admixture model with correlated allele frequencies between populations was applied without a priori information on population origin. Pilot studies with a burn-in length and MCMC (Markov chain Monte Carlo) of 100,000–300,000 were performed. Finally, 500,000 burn-ins and 500,000 iterations with ten runs were carried out to quantify the variation in the likelihood for each K. The range of possible Ks tested was 1–10. To determine the optimal number of clusters (K), an ad hoc statistic ΔK [57] was used and implemented in Structure Harvester ver. 0.6.94 [58] software set to the default parameters [59]. Additionally, principal coordinates analysis (PCoA), based on the matrix of Euclidean distances between individuals from both analysed *C. polonica* populations, was applied to investigate the genetic structure of the samples. PCoA was performed in PAST software 2.17 [60].

Finally, the bottleneck hypothesis for both populations was tested using Bottleneck software 1.2.02 [61]. To study this phenomenon using dominant AFLP markers, the infinite allele model (IAM) was used to test the mutation drift vs. bottleneck hypothesis [62]. The significance of the potential bottleneck was estimated using the sign test, the standardised mean differences test, and the one-tailed Wilcoxon signed rank test for heterozygosity excess.

## 3. Results

AFLP profiling allowed the identification of 364 bands shared among populations generated by eight primer pair combinations. The number of bands identified by individual primer pairs ranged from 34 to 56. All primer pairs generated polymorphic fragments, and their number varied from 6 to 24, for a total of 93. Most bands were present with a frequency higher than 5% within the given population. One unique band was observed in the population from the Botanical Garden (BG), while two were observed in the population from Centuria (C). The percentage of polymorphic bands in population C was higher than in the BG population (Table 1). The mean He was significantly lower in the ex situ population (mean = 0.0213) than in the Centuria population (mean = 0.0368). A permutation test (10,000 iterations) confirmed that this difference was statistically significant (*p* = 0.0371), indicating reduced allelic variation in the ex situ group. The average Shannon’s Information Index value was also significantly lower in the Botanical Garden population (mean = 0.0306) compared to the Centuria River population (mean = 0.0639). A permutation test (10,000 iterations) confirmed that this difference was statistically significant (*p* = 0.0369), supporting the observed loss of genetic variability in the ex situ group.

AMOVA revealed that 57% of the genetic variation occurred among the studied populations, whereas the remaining 43% was partitioned within populations (Table 2).

High genetic variation between populations was consistent with the F*_ST_* value and Φ*_PT_*. Nei’s genetic distance was calculated to estimate genetic differentiation between *C. polonica* populations and was 0.059 (Table 3).

**Figure 2:**
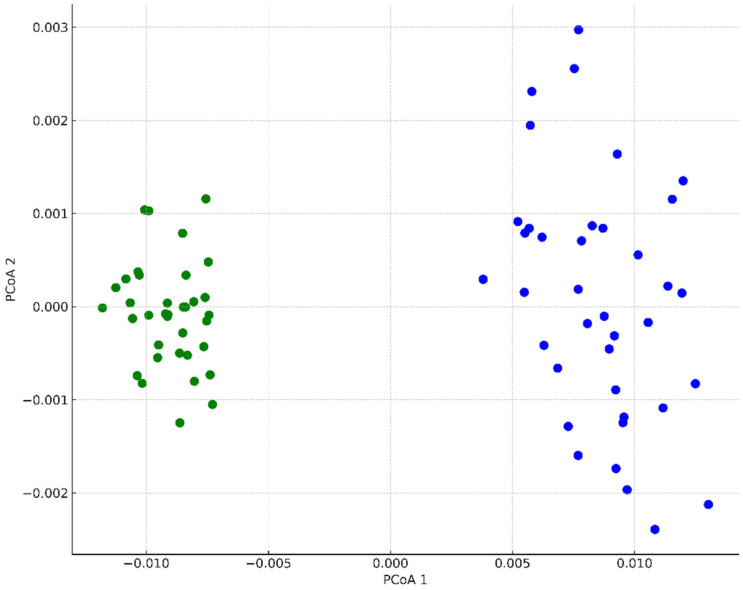
Principal coordinate analysis (PCoA) of *C. polonica* individuals representing the Botanical Garden population (green dots) and Centuria River population (blue dots).

The PCoA revealed the presence of two groups of data. The groups correspond with their origin. Individuals from the BG shared the highest degree of similarity and formed dense conglomerates, whereas those from location C were dispersed (Figure 2).

Exploiting results from Structure ver. 2.3.4 software, following the ΔK value evaluated via the Bayesian assignment, revealed the presence of a genetic structure that correlated with the analysed samples’ origin (K = 2).

Tajima’s D test was positive but insignificant for both populations. Fu’s F*s* test produced a significantly negative result for both populations, consistent with population expansion (Table 4).

The hypothesis that the observed data fit the sudden demographic/spatial expansion model was tested using the sum of square deviations (SSD) [63] and the raggedness index [64]. Here, nonsignificant values for SSD showed that the data did not deviate from that expected under the demographic/spatial expansion model. Furthermore, nonsignificant raggedness values also indicated population expansion (Table 5).

All tests produced significant *p*-values based on the IAM for both populations, with the value of particular indices being higher for the seminatural population (Table 6).

## 4. Discussion

This study assessed retrospective genetic variation in the middle lifespan of an already extinct ex situ population of *C. polonica* for the first time. Ex situ conservation measures failed after 20 years of cultivation of the population in the PAS Botanical Garden CBDC due to genetic constraints. The comparative analysis of the genetic profile included the “10-year-old” ex situ population and the “30-year-old” seminatural population. As demonstrated by the results of PCoA (Figure 2) and STRUCTURE, coefficients of Nei’s genetic distance, and AMOVA, the two populations differed significantly and were grouped according to their origin. We found that the ex situ population suffers from a significant loss of genetic diversity (decreased number of polymorphic markers and decreased heterozygosity), suggesting that this population is affected by strong demographic or selection processes. This situation is probably caused by demographic expansion following most likely the founder effect, which is in agreement with historical data, according to which the population in the Botanical Garden was established from the limited number of individuals transplanted from the seminatural site near the Centuria River. The ex situ population recovered to ca. 100 plants from this bottleneck, reached the middle of its lifespan (during sampling for this study), and firmly declined in abundance within the next ten years.

The genetic profiles of the *C. polonica* populations revealed clear and strong differentiation. This was reflected in several genetic diversity metrics. The ex situ population exhibited substantially lower genetic diversity than the seminatural Centuria River population. Specifically, both Shannon’s Information Index (I = 0.038) and expected heterozygosity (He = 0.026) were nearly two times lower than in the Centuria population (I = 0.063; He = 0.043), suggesting a reduced allelic richness and heterozygosity in the cultivated group.

AFLP profiling identified 364 fragments, with 93 polymorphic bands generated by eight primer combinations. Although both populations showed some degree of polymorphism, the Centuria population had a higher proportion of polymorphic bands (11.54%) compared to the Botanical Garden population (7.42%), and two unique bands were present only in the natural population, further emphasising its higher diversity. AMOVA supported this pattern, showing that 57% of the total genetic variation occurred between populations and only 43% within them, a finding consistent with the high F*_ST_* and Φ*_PT_* values (both = 0.572), indicating limited gene flow and long-term separation.

Principal coordinate analysis (PCoA) and Bayesian clustering (STRUCTURE) further confirmed the separation of both populations, with BG individuals forming a tight cluster and Centuria River individuals displaying a broader genetic spread. This pattern suggests a more homogeneous and possibly bottlenecked gene pool in the ex situ population, contrasted with the more structured and diverse seminatural population.

Despite initial demographic recovery, the ex situ population appears to have undergone a loss of genetic variability over time. This interpretation is supported by the results of the bottleneck tests, which indicated significant heterozygosity excess in both populations under the infinite alleles model (IAM), with stronger signals in the Centuria River population. The STRUCTURE and mismatch distribution analyses also confirmed expansion patterns, but with limited genetic input over time in the ex situ group. Fu’s F*_S_* test yielded significantly negative values in both populations, consistent with past population expansion, yet the lower diversity in the Botanical Garden group suggests that this expansion occurred from a few genotypes.

Although the ex situ population reached a high abundance (up to 100 individuals), the limited number of founding genotypes, the absence of gene flow, and possibly unintentional selection in cultivation likely contributed to genetic homogenisation. Consequently, the population declined and ultimately went extinct after just 5–6 generations, demonstrating that demographic recovery does not necessarily equate to genetic recovery.

These findings highlight the challenges of maintaining long-term genetic viability in small, isolated ex situ populations and support the need for carefully designed conservation strategies that preserve the genetic structure and avoid severe bottlenecks at establishment.

In small and isolated populations such as the ex situ *C. polonica* collection in the Botanic Garden, genetic drift is expected to lead to a gradual loss of genetic variation, increased homozygosity, and inbreeding, potentially resulting in reduced fitness. When combined with the relaxation of natural selection and the accumulation of mildly deleterious mutations, these processes may compromise long-term viability. While direct measures of fitness were not performed, the observed extinction of the ex situ population within approximately ten years after the material was sampled suggests that genetic factors likely played a major role in its decline. This corresponds to roughly 5–6 generations, a time frame within which the effects of limited genetic diversity could become pronounced under continued isolation.

This is not entirely unexpected, as other work has also shown that such adverse effects of a small population size on offspring fitness have already been found for many endangered and threatened plant species cultivated in botanical gardens (e.g., [65,66]). Consequently, ex situ populations may become maladapted to conditions in the wild, which may adversely affect their suitability as sources for reintroduction or even genetic rescue of those populations from which they were established [67,68]. Thus, inappropriate ex situ conservation can cause changes in the genetic features and traits of the population, which limits the conservation value of plant material for reintroduction purposes. Moreover, as representatives of the *Cochlearia* genus are wind-pollinated and outcrossed [22,69], there is a potential risk of hybridisation with other species of the same genus growing in botanical garden collections. Thus, the Botanical Garden plants could be unsuitable for wild population reinforcements or introduction to a new habitat. However, these risks do not negate the conservation potential of ex situ collections; rather, they underscore the importance of applying best practices in the management of living collections. To maintain their long-term conservation value, ex situ populations should be established from a representative sample of the source population, with sufficient numbers of founders to capture allelic diversity. Regular genetic monitoring, avoidance of inbreeding, and careful record-keeping of provenance and reproductive history are essential to prevent unintended genetic drift or selection. Additionally, to reduce the risk of hybridisation in outcrossing species such as *Cochlearia polonica*, spatial or reproductive isolation from congeneric taxa within botanical gardens is recommended. Where feasible, simulating environmental conditions similar to the species’ natural habitat may help mitigate maladaptation to artificial conditions. Implementing these strategies can improve the genetic integrity of ex situ collections and enhance their suitability for future reintroduction or reinforcement efforts [32]. However, such results do not entirely detract from the value of the *C. polonica* collection in the Botanical Garden. Living plants maintained in such ex situ collections are still an essential resource for educational and research purposes, but cultivation specifically for conservation is demanding and only rarely practised effectively [70].

When faced with these problems, the question arises of whether *C. polonica* can survive in a secondary site. If we look at the genetic data for a much larger population of *C. polonica* from the secondary site, the traces of the same demographic processes are visible. A positive Tajima’s D indicates a bias towards intermediate-frequency alleles, but it was not statistically significant. Fu’s F*_S_* test, which is based on the distribution of haplotypes, showed negative values, indicating an excess of rare haplotypes that would be expected under neutrality (Table 4). Thus, the hypothesis of natural evolution was significantly rejected. Moreover, nonsignificant values for the SSD raggedness index indicate population expansion, most likely following the founder effect (or the bottleneck effect, Table 5).

Additionally, all three tests (The Sing, Standardised, and Wilcoxon) used in this study analyse bottleneck effects in populations that develop transient heterozygosity excess. If the loci evolve in a strict one-step mutation model, heterozygosity excess and deficiency can occur depending on locus variability and the time elapsed since the beginning of the bottleneck. All tests produced significant *p*-values based on the IAM for both populations, indicating significant traces of bottleneck effects (Table 6). Thus, the genetic data gathered here for *C. polonica*, which showed the low genetic diversity of the ex situ population and its extinction within ten years, indicate that the “genetic health” of the species is compromised, which may decrease its adaptive capacity and threaten its survival in the wild shortly.

## 5. Conclusions

Our comparative analysis of the *Cochlearia polonica* genetic structure ex situ and that of its source population significantly contributes to the conservation management of this endemically extinct wild species represented by only one (seminatural) population. This study confirms the evidence of a recent founder effect for both populations. The seminatural population exhibited twice as much genetic diversity as the ex situ population after reaching the maximum recovery in size. Such an experience could be readily incorporated to improve the conservation management of the seminatural population in the Centuria River. It is well established that species with isolated and small populations are subject to loss of genetic diversity and a higher degree of inbreeding. Generally, fitness reduction because of loss of genetic variability increases significantly under stress [71,72]. This phenomenon makes populations much more sensitive to environmental changes, meaning they struggle to cope with rapidly deteriorating environmental conditions and significantly increasing the extinction risk [71]. The effects of genetic erosion and loss of adaptive variation are not independent. When a population is subjected to novel environmental stress, the selection intensity will increase, and the growth rate of the population will decrease. It will often become negative initially, leading to a decrease in population size, which reaches a critically low level because of selection [73]. This trend could be reversed only after adapted individuals have reached a sufficiently high frequency in the stressed population [74]. In the presence of increased inbreeding depression under stress, the progressive reduction in individual fitness is expected to reduce population numbers and growth rates much further, making adaptive recovery more difficult and severely increasing the extinction probability of such small populations. Thus, our estimates of the genetic profile of both populations are not optimistic for the seminatural population in the Centuria River since it remained an “island” population. Even with the protection of remaining habitats, further loss of genetic diversity and other genetic signatures of a bottleneck likely due to the population’s small siz and lack of gene flow and the increasing pressure of climate change, which can rapidly alter habitat conditions. Therefore, collecting seeds from the seminatural population in the Centuria River for long-term conservation through cryobanking is of the most significant importance to maintaining the current level of species’ genetic diversity. A cryopreservation protocol for *Cochlearia polonica* has already been established and successfully implemented at the PAS Botanical Garden in Powsin. Under this protocol, seeds are desiccated to an optimal moisture content and stored in liquid nitrogen at –196 °C, ensuring their long-term viability. Preliminary tests have confirmed successful regeneration after thawing, demonstrating the protocol’s effectiveness as a safe backup strategy for ex situ conservation of this critically endangered species [75].

For the seminatural population from the Centuria River, the pivotal action is the reduction in ecological and environmental factors, which may confound the genetic effects. It is essential to increase the population size to a level at which the loss of genetic diversity is minimised, either by expanding the habitat area or by enhancing the habitat quality. This action would decrease genetic risks even further and, in addition, make the population more resistant to environmental stochasticity [76].

There is also a need to improve ex situ conservation practices related to living plant collections in botanical gardens. For example, establishing ex situ populations with a large number of individuals and maintaining substantial population sizes could help preserve genetic diversity and reduce the effects of genetic drift and inbreeding [77,78]. This may also incorporate assisted ex situ conservation in botanical gardens, such as enriching the existing population with propagated individuals (e.g., obtained from seeds collected during cultivation) to increase the population size and genetic variation. Priority should be given to securing seed material for cryopreservation as an immediate safeguard, while efforts to enhance the living collection and in situ protection can proceed as part of a medium- to long-term conservation strategy.

Collecting seeds across multiple years from the seminatural population in the Centuria River for long-term conservation in the cryobank, enhancing the living plant collection in the botanical garden, and actively protecting the seminatural population in the Centuria River can synergistically act to improve the conservation management of *Cochlearia polonica* and prevent its extinction in the future.

## Figures and Tables

**Table 1:** Population genetic characteristics for the analysed populations of *Cochlearia polonica*.

Population	N	Ne	Nu	Np	P [%]	I	He
Botanical Garden	36	1.044	1	27	7.42%	0.038	* 0.026
Centuria	38	1.078	2	42	11.54%	0.063	** 0.043

N (number of samples); Ne (number of effective alleles); Nu (number of unique bands), Np (number of polymorphic bands); P [%] (percentage of polymorphic alleles)—5% criterion; I (Shannon’s Information Index); He (expected heterozygosity). * The difference in He between populations was statistically significant (*p* = 0.0371), ** the difference in Shannon’s Information Index (I) was statistically significant (*p* = 0.0369).

**Table 2:** Partitioning of diversity found in *Cochlearia polonica* from both analysed populations using AMOVA.

Source of Variation	d.f.	SS	Variance Components	Percentage of Variation
Among populations	1	275.810	7.312	57.19
Within populations	72	394.149	5.474	42.81
Total	73	669.956	12.786	

Significance test (1023 permutations).

**Table 3:** Genetic distance between populations.

Pairwise F*_ST_*	Φ*_PT_*	Nei GD	Nei Unbiased Genetic Identity
0.572	0.572	0.059	0.943

**Table 4:** Arrangements of the neutrality tests for both populations of *Cochlearia polonica*.

Test	Description	Populations		Mean	SD
		BG	C		
Tajima’s D test	S	27	42	34.50	10.607
	P_i_	7.962	13.774	10.868	4.110
	Tajima’s D	0.7733	1.343	1.058	0.403
	Tajima’s D *p*-value	0.835	0.947	0.891	0.079
Fu’s F*_S_* test	Theta p_i_	7.962	13.774	10.868	4.110
	Exp. no. of alleles	14.024	18.611	16.317	3.244
	F*_S_*	−24.921	−24.379	−24.650	0.383
	F*_S_ p*-value	0.000	0.000	0.000	0.000

**Table 5:** Estimates of mismatch analysis of the studied *Cochlearia polonica* populations.

Model	Statistic	Population		Mean	SD
		BG	C		
Demographic expansion	SSD	0.005	0.001	0.003	0.003
	Model (SSD) *p*-value	0.150	0.740	0.445	0.417
	Raggedness index	0.013	0.005	0.009	0.006
	Raggedness *p*-value	0.230	0.600	0.415	0.262
Spatial expansion	SSD	0.005	0.001	0.003	0.003
	Model (SSD) *p*-value	0.140	0.620	0.380	0.339
	Raggedness index	0.013	0.005	0.009	0.006
	Raggedness *p*-value	0.340	0.560	0.450	0.156

**Table 6:** Testing bottleneck versus mutation drift equilibrium hypotheses for analysed populations of *Cochlearia polonica*.

Population	SIGN Test	Standardised Test	Wilcoxon Test
Botanical Garden	Heex = 11.96 Hd = 5 Hex = 22 *p* = 0.00009	T2 = 3.801 *p* = 0.00007	One tail for heterozygosity deficiency: 0.99976 One tail for heterozygosity excess: 0.00027 Two tails for heterozygosity excess and deficiency: 0.00054
Centuria	Heex = 18.66 Hd = 7 Hex = 35 *p* = 0.0000	T2 = 5.713 *p* = 0.0000	One tail for heterozygosity deficiency: 1.0000 One tail for heterozygosity excess: 0.0000 Two tails for heterozygosity excess and deficiency: 0.0000

Abbreviations: Heex—expected number of loci with heterozygosity excess; Hd—number of loci with heterozygosity deficiency; Hex—number of loci with heterozygosity excess.

## Data Availability

The data supporting the findings of this study are available in the Supplementary Materials of this article.

## Supplementary Materials

The following supporting information can be downloaded at https://www.mdpi.com/article/10.3390/biology14060681/s1, Table S1. Biol_Supplementary Cochlearia polonica_data_matrix; Table S2. Sequences of adapters, preselective, and selective primers used for AFLP analysis.
