# Supplementary material for: Halfway Through Ex Situ Population Genetic Lifespan: The Case of Cochlearia polonica
Source: Biology (Basel). 2025 Jun 11;14(6):681. doi: 10.3390/biology14060681 (PMC12189762; doi:10.3390/biology14060681)
Supplement: Supplementary file 1 [file biology-14-00681-s001.zip › Biol_Table_S2_Sequences of primers for AFLP analysis.pdf]

Table S2. Sequences of adapters, preselective and selective primers used for AFLP analysis

Type	Sequence (5′→ 3′)
Adapters *Eco*RI	CCATGCGTCAGATGCTC
	CATCTGACGCATGGTTAA
Adapters *Mse*I	TACTCAGGACTCATA
	GACGATGAGTCCTGAG
*Eco*RI preselective primer	GACTGCGTACCAATTCA
*Mse*I preselective primer	GATGAGTCCTGAGTAAC
*Eco*RI selective primer	GACTGCGTACCAATTCAxx
*Mse*I selective primer	GATGAGTCCTGAGTAACxx

Adapter and primer sequences; xx – any combination of the nucleotides at the primers 3’ ends
